# Olfactory Neuroblastoma of the Sinonasal Tract with Prominent Orbital Protrusion: A Case Report and Literature Review

**DOI:** 10.1007/s12070-020-02359-x

**Published:** 2021-01-27

**Authors:** Lin Chen, Jingxin Wang, Zhigang Yang, Yingkun Guo

**Affiliations:** 1 Department of Radiology, West China Second University Hospital, Sichuan University, Key Laboratory of Obstetric and Gynecologic and Pediatric Diseases and Birth Defects of Ministry of Education, 20# Renmin Road, Chengdu, 610041 Sichuan China; 2 Department of Ultrasound, West China Second University Hospital, Sichuan University, Key Laboratory of Obstetric and Gynecologic and Pediatric Diseases and Birth Defects of Ministry of Education, 20# Renmin Road, Chengdu, 610041 Sichuan China; 3 Department of Radiology, West China Hospital, Sichuan University, 37# Guo Xue Xiang, Chengdu, 610041 Sichuan China

**Keywords:** Olfactory neuroblastoma, Magnetic resonance imaging, Computed tomography, Pathology

## Abstract

Olfactory neuroblastoma (ONB) is a rare malignant neuroectodermal tumor of the nasal cavity. Olfactory neuroblastoma centered in the posterior right orbit with prominent orbital protrusion is even rare. Grading ONB is extremely important as individualized treatment plans must be formulated according to tumor grade. We report the case of a 67-year-old female who presented with the chief complaints of persistent nasal congestion with intermittent epistaxis and unilateral proptosis over the past five years. Radiological imaging was suggestive of a large heterogeneous mass in the right superior nasal cavity with extensions into the right medial orbit, nasopharynx, the right maxillary sinus, the anterior cranial fossa, right ethmoidal, frontal and bilateral sphenoidal sinuses, as well as into the right frontal lobe. Assessment of the radiologic features revealed the diagnostic possibility of olfactory neuroblastoma. A nasopharyngeal biopsy confirmed an olfactory neuroblastoma. Frontal osteoplastic craniotomy and excision of the intracranial part of the tumor from above and transnasal endoscopic removal of the mass in the nasal cavities, paranasal sinuses and right medial orbit from below was done. Evaluation of histopathological characteristics and immunohistochemical findings revealed a diagnosis of WHO grade IV olfactory neuroblastoma. Because of poor economic condition, the patient did not take adjuvant radiotherapy and chemoradiation and post-operative examination. We report a huge ONB centered in the posterior right orbit with prominent orbital protrusion. Magnetic resonance image and computed tomography are helpful for evaluating the appearance and the extent of ONB, as well as grading this tumor, which may aid therapeutic decisions and improve survival.

## Background

Olfactory neuroblastoma (ONB), also called as esthesioneuroblastoma, is a rare malignant neoplasm of the nasal cavity arising from the olfactory neuroepithelium. It accounts for 3–5% of all nasal and sinonasal malignancies with no race or gender predilection [1]. ONB has a bimodal distribution and tends to occur around the second and sixth decade of life [2]. It has an incidence of 0.4 per million of population and ONB centered in the posterior right orbit with prominent orbital protrusion is even rare. Keeping in mind the rarity of ONB and the diagnostic difficulty, we present a unusual case of ONB with prominent orbital protrusion and discuss the differentiation from other nasal cavity neoplasms as emphasized in the literature review.

## Case presentation

### History

A 67-year-old female was presented to the Otolaryngology department with unilateral prominent orbital protrusion and a mass in the right nasal cavity, which caused persistent nasal congestion with intermittent epistaxis over 5 years. Three years earlier she had been treated for suspected chronic rhinitis without resolution of symptoms. She came for further treatment with progressive enlargement of the mass and worsening of symptoms over the last 2 years.

### Clinical Exam

A nasal exam was performed using rigid endoscopy, which revealed a large mass, medial to the middle turbinate in the right nasal cavity. The mass extended to the choana, but did not appear to extend into the nasopharynx clinically. The lesion was pink and lobulated with a rubbery, non-friable texture. No lesions were identified in the left nasal cavity. Examination of visual acuity revealed no visual impairment. She had no history of headache, dizziness, or diplopia. Her blood profile for biochemistry and hematology was within normal limits. Tests for human immunodeficiency virus (HIV), hepatitis B surface antigen (HBsAg), and hepatitis C virus were negative.

### Radiographic Features

**Fig. 1:**
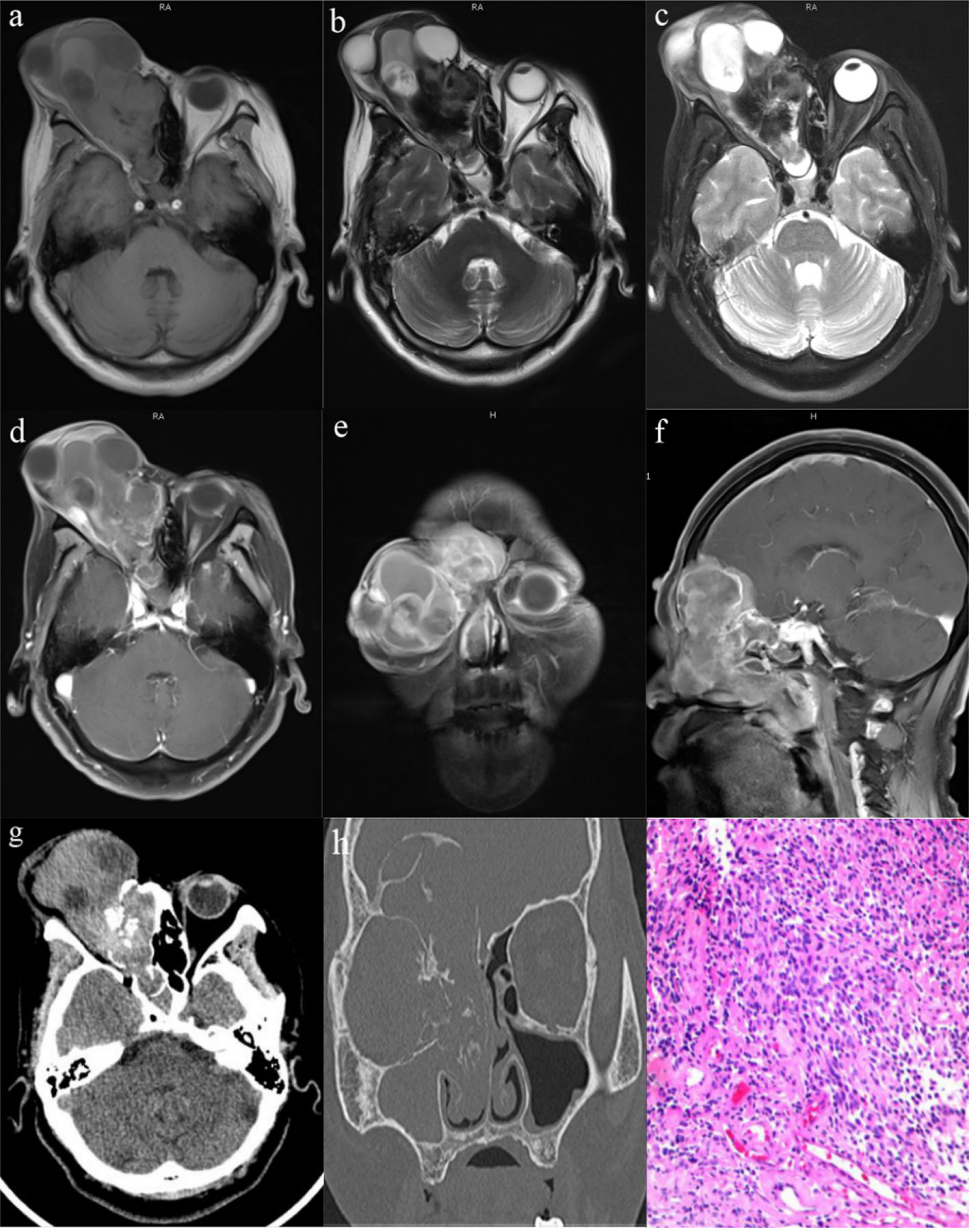
Pre-operative contrast-enhanced MRI and CT scan of the brain and paranasal sinuses and the histopathological examination with hematoxylin and eosin stained. **a**–**c** Non-contrast MRI revealed heterogeneous isointense signal of the mass on T1-weighted sequences and hypointense to hyperintense signal on T2-weighted sequences and fat attenuated sequences, with unclear boundaries and large invasion range. **d**–**f** Contrast-enhanced MRI showed dumbbell shaped mass with intense heterogeneous post contrast enhancement in the right superior nasal cavity with extensions into the right orbit, anterior cranial fossa and paranasal sinuse. Peritumoral cysts are also noted at the tumor brain interface. **g**, **h** CT scan of the brain and paranasal sinuses revealed that there was erosion of the right paranasal sinuses wall, the cribriform plate, and the right medial orbital wall with significant bony destruction and radial periosteal reaction. The ‘waist’ of the dumbbell shaped mass is at the cribriform plate. **i** Hematoxylin and eosin stained slide (× 200)—showing olfactory neuroblastoma which is having a high nuclear: cytoplasmic ratio, round hyperchromatic nuclei with inconspicuous nucleoli, scanty cytoplasm, and a richly intercellular vascularized fibromyxoid stroma

Magnetic resonance image (MRI) studies revealed a heterogeneous mass with isointense on T1-weighted imaging (Fig. 1a), isointense to hyperintense on T2-weighted imaging (Fig. 1b), and hyperintense on fat attenuated imaging (Fig. 1c). The mass was heterogeneous contrast-enhanced and centered in the posterior right orbit, with 57 × 94 × 91 mm in size (Fig. 1d). The lesion was extending laterally into the right medial orbit and the right maxillary sinus with blockage of the osteomeatal complex (Fig. 1e), medially into the right nasal cavity with slight deviation of the nasal septum to the left side, and posteriorly into the nasopharynx (Fig. 1f). Superiorly, the lesion was seen to erode the cribriform plate and extend into the anterior cranial fossa, as well as into the right frontal lobe. There was evidence of peritumoral cysts at the tumor-brain interface with perilesional edema. The lesion involved right ethmoidal, frontal and bilateral sphenoidal sinuses also. Additionally, there was mucosal thickening in the left frontal sinus. No pathologically enlarged retropharyngeal or cervical lymph nodes were noted.

The computed tomography (CT) scans demonstrated a heterogeneously enhancing lobular soft tissue mass that filled the right nasal cavity and orbit (Fig. 1g). Contrary to the clinical impression, the mass did extend posteriorly into the right nasopharynx, causing bowing and thinning of the right maxillary sinus wall. There was erosion of the cribriform plate and crista galli with further invasion into the right frontal sinus and the right medial orbital wall (Fig. 1h). No calcifications were identified in the mass. The left frontal and maxillary sinuses revealed mucosal thickening. There were numerous small unremarkable cervical lymph nodes with no pathologic enlargement.

### Histological Characteristics and Immunohistochemical Findings

Frontal osteoplastic craniotomy and excision of the intracranial part of the tumor from above and transnasal endoscopic removal of the mass in the nasal cavities, paranasal sinuses and right medial orbit from below was done. On histopathological examination, evaluation of the hematoxylin and eosin stained biopsy specimen revealed interconnecting tumor lobules embedded within a richly vascularized fibromyxoid stroma (Fig. 1i). The tumor was composed of lobules, sheets, and nest of primitive cells which were displaying high nuclear:cytoplasmic (N:C) ratio, pleomorphism, round hyperchromatic nuclei with inconspicuous nucleoli, and scanty cytoplasm. On immunohistochemistry, the tumor cells were immunoreactive for neuron-specific enolase (NSE), synaptophysin, chromogranin, CD56, Bcl-6, CD99, and peripherally for S100, but nonreactive for cytokeratin (CK), smooth muscle actin (SMA), and epithelial membrane antigen (EMA). The cytomorphologic and immunophenotypic features were characteristic of a WHO grade IV olfactory neuroblastoma.

## Discussion

Olfactory neuroblastoma (ONB) is a rare malignant tumor of neural crest origin, arising from the olfactory epithelium of the nasal vault [3]. It is a relatively uncommon neoplasm, and its clinicopathological presentation remains unclear. Berger and Luc first described this uncommon neoplasm in 1924. Since then, approximately 1200 cases of ONB have been identified [2]. However, ONB centered in the posterior right orbit with prominent orbital protrusion reported in this case is even rare. This disease has a higher degree of malignancy, and the younger the age, the worse the prognosis. Given the pathway of the olfactory nerve, ONB can possiblely invade adjacent structures, such as the ethmoidal sinus, anterior skull base, orbit, and even across the midline to the contralateral nasal cavity [1]. If metastatic, ONB may involve local lymph nodes, with distant metastasis to lungs, liver, and bone.

The most common presenting symptoms are unilateral nasal obstruction (70%), and epistaxis (50%). Other symptoms include anosmia, headache, pain, excessive lacrimation, and rhinorrhea. In particular, patients with periorbital extension may present with proptosis, periorbital edema and decrease visual acuity, just like the rare case we reported. Uncommonly, ONB may be associated with syndrome of inappropriate antidiuretic hormone secretion (SIADH) with dilutional hyponatremia or ectopic adrenocorticotropic hormone (ACTH) production leading to Cushing syndrome [4].

ONB may histologically mimic a number of types of tumor within the sinonasal tract, making it much difficult to diagnosis [5]. Multi-modality imaging is essential to correctly assess the extent of the disease in the management of this infrequent tumor [6]. CT and MRI with and without contrast is the first line for evaluation of ONB. The imaging pathway in this case was typical, with CT and MRI complementing each other in maximizing tumor delineation [7]. The CT scan is a helpful initial study to identify the lesion but more importantly, it has superior definition in reviewing bony involvement of the cribriform plate, orbit, and air sinuses [3]. Typically, on CT, a homogenous mass with necrotic nonenhancing areas is observed. As to MRI, ONB usually appears hypointense on T1-weighted sequences and intermediate to hyperintense on T2-weighted sequences. Contrast enhancement is avid and homogeneous, except for areas of necrosis or hemorrhage. MRI has superiority in evaluating the extent of soft tissue invasion and establishing tumor boundaries against post obstruction fluid in the paranasal sinuses. Typically, a dumbbell shaped mass extending across the cribriform plate is one of the most characteristic findings of this tumor [8]. The upper portion of the dumbbell-shaped mass is in the anterior cranial fossa whereas the lower portion is in the nasal cavity with the waist at the cribriform plate [9]. Another characteristic imaging feature of ONB is the presence of peritumoral cysts at the tumor brain interface [2]. In this case, we can also see a dumbbell-shaped mass with the waist at the cribriform plate and peritumoral cyst, which is helpful in the accurate diagnosis of ONB.

Histologically, ONB typically have a high nucleus/cytoplasm ratio, with scant, poorly defined cytoplasm. High-grade tumors characteristically present with nuclear pleomorphism, mitotic figures (> 2 per high-power microscope field), and necrosis [10]. Homer Wright rosettes are present in up to 30% of cases and Flexner–Wintersteiner rosettes are seen in up to 5%. The ultrastructural features of ONB are dense membrane-bound neurosecretory granules with neurofilaments and micro-tubules in the cytoplasm and nerve processes. They typically stain positive for NSE, neurofilament protein, synaptophysin, chromograinin, CD56, and LEU-7. S-100 protein staining seen along the periphery of the neoplastic lobules, also seen in our case, is the characteristic manifestation of ONB. Hematolymphoid markers (CD45, B cell, and T cell), myogenic markers (myoglobin, desmin, myogenin), melanoma markers (HMB 45, melan A, tyrosinase) and Ewing’s sarcoma markers (CD99/MIC2) are absent in most ONB cases. Epithelial markers including EMA, SMA and carcinoembryonic antigen (CEA) are also absent in ONB.

The diagnosis of ONB is extremely challenging as several sinonasal neoplasms must be excluded. The differential diagnosis is widely broad including rhabdomyosarcoma, lymphoma, undifferentiated carcinoma, neuroendocrine carcinoma, melanoma and Ewing’ s sarcoma [11]. The differential diagnosis of ONB and neuroendocrine carcinoma was considered as ONB is non-reactive with CK and TTF-1. ONB can be separated from Ewing’ s sarcoma by the presence of an S100 positive sustentacular network in most cases as well as diffuse positivity for neuroendocrine markers and negativity for CD99 [12]. But untypically in this case, the tumor cells were positive for CD99. In addition, the maximum standardized uptake value in the initial PET/CT may be an adjunct to the differential diagnosis of ONB and sinonasal undifferentiated carcinoma [13]. Furthermore, magnetic resonance diffusion kurtosis imaging (DKI) and dynamic contrast enhanced MRI (DCE-MRI) are helpful in distinguishing ONB from nasal squamous cell carcinoma for significantly higher K values and lower V_e_ values in ONB [1]. In particular, ONB can be differentiated from intracranial immature teratoma for the absence of some increased substances including AFP, b-HCG, and PLAP in serum and cerebrospinal fluid [14]. The diagnosis of ONB can be established after a careful evaluation of radiological study, histopathological examination, immunohistochemistry, and cytogenetic analysis [11].

Kadish et al. and Dulguerov et al. proposed ONB classifications based on primary tumor extension and clinicoradiographic data, respectively. Kadish et al. were the first to propose a staging classification for ONB [8]. Morita et al. modified the Kadish staging to include 4 groups. The modified Kadish staging classification is the most commonly used approach to classify the anatomic extent of the tumor (Table 1). According to modified Kadish staging, our case belongs to group C. The Dulguerov system (Table 2), which uses the TNM classification and includes the imaging data, is preferred by some oncologists and surgeons for recognizing the early involvement of the cribriform plate in the T2 stage and identifying the true brain involvement. ONB is divided into 4 grades by Hyams based on the following microscopic features: cellular architecture and pleomorphism; mitotic activity; and presence of necrosis, calcification, gland proliferation, and neurofibrillary matrix or rosettes [15]. Histopathology is considered a potentially important prognostication, Hyams grades III and IV are associated with a poor prognosis.

**Table 1:** Modified Kadish staging classification and Hyams histologic grading system for olfactory neuroblastoma

Staging	Description
*Modified Kadish classification*	
A	Tumor confined to the nasal cavity
B	Tumor extension to the paranasal sinuses
C	Tumor beyond the nasal cavity and paranasal sinuses, including involvement of the cribriform plate, base of the skull, intracranial cavity, and/or orbit
D	Tumor with metastases to cervical lymph nodes and/or distant sites

Grade	I	II	III	IV
*Hyams histologic grading system*				
Cytoarchitecture	Lobular	Lobular	±	±
Mitotic rate	0	Low	Moderate	High
Nuclear Pleomorphism	Absent	Slight	Moderate	Marked
Rosettes	±	±	True Rosettes	None
Necrosis	Absent	Absent	Mild	Extensive

± , may be present or absent

**Table 2:** The Dulguerov staging system for olfactory neuroblastoma

Staging	Description
*Primary tumor*	
T1	Tumor involving the nasal cavity and/or paranasal sinuses (excluding sphenoid), sparing the most superior ethmoid
T2	Tumor involving the nasal cavity and/or paranasal sinuses (including the sphenoid) with extension to or erosion of the cribriform plate
T3	Tumor extending into the orbit or protruding into the anterior cranial fossa, without dural involvement
T4	Tumor involving the brain
*Lymph nodes*	
N0	No cervical lymph node metastasis
N1	Any form of cervical lymph node metastasis
*Distant metastasis*	
M0	No metastases
M1	Distant metastases

The management of ONB usually requires craniofacial surgical approach, trephination procedure, which is technically challenging and achieving good results are difficult. Recently, authors have come to an agreement that a multimodal approach is necessary to treat patients with ONB. Treatment modalities for ONB are enbloc resection, extra cranial resection or surgery combined with radiotherapy and/or chemotherapy. But unfortunately, the patient did not take adjuvant radiotherapy and chemoradiation and post-operative examination because of poor economic condition.

## Conclusions

We report a huge ONB uncommonly centered in the posterior right orbit with prominent orbital protrusion. From an analysis of the findings in the olfactory neuroblastomas, the diagnosis of ONB should be considered when radiographic imagings reveal a dumbbell-shaped mass in the nasal cavity with peritumoral cyst, and multi-modality imaging are helpful for evaluating the appearance and the extent of ONB, as well as grading this tumor, which may aid therapeutic decisions and improve survival.

## Data Availability

The data that support the findings of this study are available from the corresponding author upon reasonable request.

## Abbreviations

ONB: Olfactory neuroblastoma
MRI: Magnetic resonance image
CT: Computed tomography
HIV: Human immunodeficiency virus
HBsAg: Hepatitis B surface antigen
N:C: Nuclear:cytoplasmic ratio
NSE: Neuron-specific enolase
CK: Cytokeratin
SMA: Smooth muscle actin
EMA: Epithelial membrane antigen
SIADH: Syndrome of inappropriate antidiuretic hormone secretion
ACTH: Adrenocorticotropic hormone
CEA: Carcinoembryonic antigen
DKI: Magnetic resonance diffusion kurtosis imaging
DCE-MRI: Dynamic contrast enhanced MRI
